# An Approach for the Estimation of Concentrations of Soluble Compounds in *E. coli* Bioprocesses

**DOI:** 10.3390/e25091302

**Published:** 2023-09-06

**Authors:** Deividas Masaitis, Renaldas Urniezius, Rimvydas Simutis, Vygandas Vaitkus, Mindaugas Matukaitis, Benas Kemesis, Vytautas Galvanauskas, Benas Sinkevicius

**Affiliations:** Department of Automation, Kaunas University of Technology, LT-51367 Kaunas, Lithuania

**Keywords:** recurrent neural networks, soft sensor, ensemble averaging, in vitro, off-gas, *E. coli*, solutes

## Abstract

Accurate estimations of the concentrations of soluble compounds are crucial for optimizing bioprocesses involving *Escherichia coli* (*E. coli*). This study proposes a hybrid model structure that leverages off-gas analysis data and physiological parameters, including the average biomass age and specific growth rate, to estimate soluble compounds such as acetate and glutamate in fed-batch cultivations We used a hybrid recurrent neural network to establish the relationships between these parameters. To enhance the precision of the estimates, the model incorporates ensemble averaging and information gain. Ensemble averaging combines varying model inputs, leading to more robust representations of the underlying dynamics in *E. coli* bioprocesses. Our hybrid model estimates acetates with 1% and 8% system precision using data from the first site and the second site at GSK plc, respectively. Using the data from the second site, the precision of the approach for other solutes was as fallows: isoleucine −8%, lactate and glutamate −9%, and a 13% error for glutamine., These results, demonstrate its practical potential.

## 1. Introduction

In recent years, *Escherichia coli* has emerged as a prominent host organism for a wide range of bioprocesses, including the production of biofuels [1], pharmaceuticals [2], and other high-value products [3]. Optimizing these bioprocesses requires precise knowledge of the concentrations of soluble compounds, which play crucial roles in shaping cellular metabolism and growth. An accurate estimation of these compound concentrations is essential for understanding and controlling the underlying dynamics of *E. coli* bioprocesses. Conventionally, chemical analysis and laboratory techniques have been used to determine soluble compound concentrations. However, these methods are often time-consuming and labor-intensive and may disrupt continuous bioprocess monitoring. Consequently, there is a growing need for alternative approaches which provide real-time estimations without sacrificing precision [4,5].In response to this demand, this paper presents a hybrid model structure that leverages off-gas data and physiological variables to estimate concentrations of diverse soluble compounds in *E. coli* bioprocesses. The proposed hybrid model utilizes recurrent neural networks, which are particularly suitable for modeling dynamic and time-dependent processes.

To improve the precision of estimations, the proposed hybrid model employs ensemble averaging combined with an entropic information gain. By incorporating varying neural network inputs, the model aims to comprehensively represent the underlying dynamics in *E. coli* fed-batch cultivations. Ensemble averaging provides additional information, leading to more robust and reliable estimations.

In this paper, Section 1 discusses the reasons for using noninvasive means for bioprocess monitoring; Section 2 reviews the literature related to the study; Section 3 describes the cultivation conditions, dataset collection, and physiological parameters; Section 4 outlines the structure of the hybrid model; Section 5 presents an experimental investigation of the hybrid model, including evaluation of model performance, determination of optimal committee size, and the precision of the model for estimating levels of acetate and other soluble compounds; Section 6 summarizes the results and outlines future uses of the model; and the Section 7 discusses the conclusions of this study.

## 2. Related Works

### 2.1. Digital Twin and Open-Loop Estimation

Early estimations in the field relied on open-loop estimation techniques. Open-loop estimators do not incorporate feedback from the system being estimated, which means that the estimates based on data measured during the estimation process. Instead, these methods rely on predetermined models or equations to predict the behavior of the system. Early examples included, flux balance analyses [6], balances of biomass concentrations and specific growth and substrate uptake rates [7,8,9,10]. To estimate various soluble compounds, these models relied on data such as the liquid volume, biomass, and glucose concentrations. In addition, all variables for the estimates were derived via differential equations, using primarily substrate feeding profiles.

As research advanced, more sophisticated approaches were explored. For example, a kinetic model [11] was developed. The extended Kalman filter (EKF) was another popular method for estimating solutes. In 2005, a digital twin was constructed using the EKF method together with an asymptotic observer [12]. Additionally, advanced methods, such as pulse-based systems [13], event-driven models [14], and adaptive Luenberger observers [15], also contributed to improving the estimates of soluble compounds.

In later years, empirical models became increasingly popular for forecasting soluble compounds. Of the various data-driven approaches, recurrent neural networks (RNN) and long short-term memory (LSTM) networks were extensively exploited for lactate estimation [16]. A notable research paper published in 2023 compared the performance of multilayer perceptron (MLP), RNN, LSTM, and gated recurrent unit (GRU) neural network models for the estimation of lactate [17]. The comparison aimed to assess the strengths and weaknesses of the architecture of each neural network architecture in order to obtain the most accurate and reliable predictions of lactate levels.

### 2.2. Closed-Loop Estimation

In the realm of closed-loop estimation, various approaches have been adopted to improve the accuracy and reliability of solute estimations. In the early years, indirect methods were commonly used to identify acetates, involving specific production rates, volume ratios [18], or dissolved oxygen probes [19]. High-performance liquid chromatography (HPLC) was also used to aid the estimation process [20].

As studies evolved, researchers began implementing the extended Kalman filter method to estimate solutes. It was applied as either a standalone estimator [21] or together with asymptotic observers [22]. By incorporating feedback from real-time data, EKF-based approaches allowed for dynamic adjustments of estimates, leading to improved predictions of solutes’ concentrations.

Empirical models emerged in 1992 with the adoption of feed-forward and recurrent neural networks to estimate ethanol [23]. However, they gained greater popularity in later years [24]. Subsequent studies employed various neural network architectures for ethanol concentration, such as the radial basis neural network (RBNN) [25]. These empirical models provided valuable insights into the relationship between input data and the estimation of soluble compounds.

Hybrid approaches combining mechanistic and data-driven metabolite estimation techniques were also explored [26]. Together with a data reconciliation algorithm [27], a model proposed by M. Dabros et al. incorporated mass balances of off-gas information, primarily oxygen and carbon dioxide concentrations. Our previous work also suggested a hybrid approach for acetate estimation using a decision tree model [28]. It incorporated off-gas information and information related to cell age and physiological variables such as the specific growth rate.

An overview of soluble compound estimation methods can be seen in Table 1.

## 3. Materials and Methods

### 3.1. Cultivation Conditions

Data for this study were gathered from two separate sites during a fed-batch process. The data acquisition process involved monitoring various parameters and measurements.

#### 3.1.1. Site 1: Recombinant *E. coli* BL21 (DE3) pET21-IFN-alfa-5 Cells

A total of 287 samples provided data from 17 different cultivation processes [29]. Of these experiments, growth-limiting feeding was used in 8 instances, with negligible glucose concentrations. The duration of each cultivation did not exceed 20 h. Real-time monitoring of the bioreactor off-gas was conducted utilizing a BlueSens gas analyzer (BCpreFerm, BlueSens) equipped with sensors for O_2_, CO_2_, and pressure measurements. The utilization of airflow data derived from the Applikon BioBundle bioreactor facilitated the comprehensive investigation of the oxygen uptake and carbon dioxide production rate. The experimental conditions at Site 1 are depicted at Table 2: 

#### 3.1.2. Site 2: BL3 (DE3) *E. coli* Strain from GSK

In addition to the previous experiments, data from four further experiments were obtained from the project “Multi-Omic Characterization of *E. coli* Cultures for Antigen Protein Production (MOCECAP)” conducted by GSK [30]. These additional experiments involved taking a total of 72 samples. A “Cedex Bio” analyzer was utilized to measure soluble compounds other than acetates, which were measured using enzymatic assay kit. Two of the four experiments produced insoluble WT1-A10 (Wilms’ tumor protein), whereas the remaining two experiments primarily produced the soluble recombinant fusion protein F4co. The duration of the cultivation processes for all the experiments conducted at this site was 66 h. The experimental conditions at Site 2 are depicted at Table 3: 

### 3.2. Dataset Collection

The dataset comprises a range of vital parameters that contribute to a comprehensive understanding of the cultivation process. These parameters encompass the temporal aspect, reflecting the duration of the cultivation process. Additionally, the initial biomass concentration provides information on the start of the cultivation process, including the onset of cell growth and metabolic activity. The oxygen uptake rate (OUR) quantifies cellular respiration, illustrating the rate at which cells consume oxygen and indicating their metabolic vitality and energy requirements. The weight of the microbial broth serves as an indicator of its overall volume.

Furthermore, the dataset incorporates information regarding the glucose substrate employed for feeding during experiments. Glucose is a primary energy source and a pivotal precursor for cellular processes. The initial concentration of glucose at the beginning of biosynthesis provides information on the primary energy source available to the cells before the start of the feeding process, including its accessibility and subsequent utilization. Site 2 was supplemented with an additional carbohydrate source, L-Isoleucine, which was exploited accordingly.

Similarly, the carbon dioxide production rate (CPR) is significant because it measures cells’ the metabolic activity of cells and their efficiency in converting substrates into desired products. The overall metabolic flux and productivity of the system can be assessed by quantifying the rate at which carbon dioxide is produced.

Finally, including the time of induction (ITPG) information in the dataset enables us to explore the temporal dynamics of the bioprocess metabolism and its correlation with the initiation of the synthesis process of target product.

The comprehensive fusion of these diverse observations contributes to the characterization of the bioprocess. This, in turn facilitates analysis of the multifaceted factors which influence biomass growth, substrate consumption, metabolic dynamics, and production of the target product.

### 3.3. Latent Parameters

For this study, we incorporate a set of nuisance parameters (*α* and *β*) to derive the latent variables [31], including biomass concentration. The biomass concentration is a variable physiological state in bioprocesses, providing crucial information on microbial growth and productivity. To accurately estimate the biomass concentration, we employed the dynamic biomass concentration assessment suggested in research by Survyla et al. [32]. The assessment considers the oxygen uptake rate (OUR) and nuisance parameters *α* and *β*, which characterize the specific cell strain [33].

The following formula determines the latent biomass concentration:

(1) $$\left\{\begin{array}{c} x\left(t_{i}\right)=x_{0}+\sum_{j=1}^{i}\frac{OUR\left(t_{j}\right)}{\alpha \left(t_{j}\right)}∆t_{j};\;\;\;\;\;\;\;\;\;\;\;\;\;\;\;\;\;\;\;\;\;\;\;\;\;\;\;\;\;\;\;\;\;\;\;\;\;\;\;\;\;\;if\;\sum_{j=1}^{i-1}X\left(t_{j}\right)∆t_{j}\leq k_{cX}\\ x\left(t_{i}\right)=\frac{x_{0}+\sum_{j=1}^{i}\frac{OUR\left(t_{j}\right)+\beta \left(t_{j}\right)\cdot X_{cX}}{\alpha \left(t_{j}\right)}e^{\sum_{k=1}^{j}\frac{\beta \left(t_{k}\right)}{\alpha \left(t_{k}\right)}∆t_{k}}∆t_{j};}{e^{\sum_{j=1}^{i}\frac{\beta \left(t_{j}\right)}{\alpha \left(t_{j}\right)}∆t_{j}}},\;\;\;\;\;\;\;\;\;\;\;\;\;\;\;\;\;\;\;\;\;\;\;\;\;\;\;\;\;\;\;\;\;otherwise, \end{array}\right.$$

where the latent biomass concentration is represented by x, OUR indicates the oxygen uptake rate, and the biomass concentration at the moment of inoculation is indicated by $x_{0}$. The parameter which describes the time at which the maintenance term *β* becomes involved in the growth model is represented by *k_cX_*. 

The latent specific growth rate (*μ*) is a crucial parameter for capturing the kinetics of cell growth in bioprocesses:

(2) $$\mu_{i}=\frac{{dX}_{i}}{dt\cdot X_{i}},$$

The specific growth rate is a characteristic of the physiological state of the cell culture and serves as an indicator of the overall quality of the cultivation process [34,35,36]. Quantification of the growth rate provides valuable insights into the metabolic activity and efficiency of the cells within the bioprocess.

Furthermore, some researchers have proposed that the kinetic parameters in bioprocesses depend on the average age of the cell population. Such an assumption suggests that these parameters are influenced not only by the immediate state of the cells but also the average age of the individual cells which are present. The latent average age points to the active biomass concentration within the population [37,38]:

(3) $$\bar{{Age}_{i}}=\frac{\sum_{j=0}^{i}\left(t_{i}-t_{j}\right)∆X\left(t_{j}\right)}{X_{i}},$$

In our study, we expanded upon this concept by incorporating the latent cumulative cell age as an additional parameter:

(4) $$\bar{{cAge}_{i}}=\sum_{j=0}^{i}\left(t_{i}-t_{j}\right)∆X\left(t_{j}\right),$$

**Figure 1:**
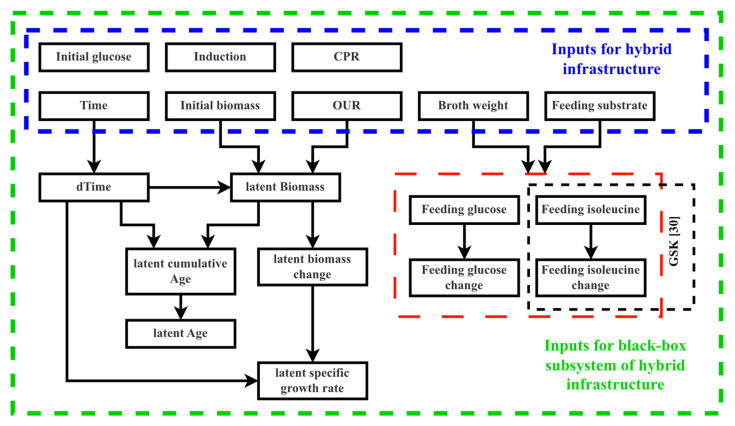
Relationships tree between the measured and derived model variables.

The cumulative cell age represents the current biomass-concentration-hours state variable. The following diagram in Figure 1, demonstrates the interconnection between the observed and derived physiological parameters.

Figure 1 presents the measured variables (indicated by the dashed blue square) and their relationships in the derivation of further variables. Feeding information (indicated by dashed red square) is dependent on the specific site conditions; although Site 1 solely employed glucose for feeding, Site 2 introduced isoleucine as a supplementary nutritional factor. Within the framework of the black box model, the inputs encompass not only the deduced parameters but also the initially gauged variables.

## 4. Recurrent Neural Network Development for the Hybrid Model

In this study, we used the long short-term memory (LSTM) modeling technique to explore the modeling of the acetate concentrations in E. coli cultivation using the Long-Short-Term Memory (LSTM) modeling technique. LSTM, a specialized recurrent neural network (RNN) architecture, has shown remarkable effectiveness in the modeling and prediction of time series data [39,40]. It was specifically designed to address the problem of vanishing gradients encountered by traditional RNNs, which restricted their ability to capture long-term dependencies in sequential data. By maintaining a constant error gradient, the memory cells within LSTMs enable the network to learn and propagate information over extended sequences. This characteristic makes LSTM particularly suitable for estimating concentrations of soluble compounds in *E. coli* bioprocesses [36].

### 4.1. Network Structure

In our study, we utilized the Keras (version 2.12.0) and TensorFlow (version 2.12.0) libraries in Python (version 3.9.7) to construct the LSTM model. The architecture consisted of an LSTM layer with 23 units and a rectified linear unit (ReLU) activation function. To mitigate overfitting, dropout layers with a rate of 0.2 were incorporated. Following the LSTM layer, dense layers consisting of 10 units and a ReLU activation function were added. An additional dropout layer with a rate of 0.2 was introduced before the final dense layer, which consisted of a single unit. To ensure the non-negativity of the output, we enforced non-negativity constraints on the kernel and bias of the last dense layer.

To measure the training process, we employed a modified mean square error (*MMSE*):

(5) $$MMSE=\frac{\sum_{i=1}^{n}{\left({|A}_{i}^{*}-A_{i}|+1\right)}^{2}}{n},$$

where $A_{i}^{*}$ is the *i*-th observation of the compound, $A_{i}$ is the hybrid model estimate, and *n* is the number of values. Mean absolute error modification ensured that errors below one unit were given appropriate importance, leading to a more balanced (unbiased) assessment of the performance of the model. We used the Adam optimizer with a learning rate of 0.001 for model training. We implemented early stopping based on the validation loss to prevent overfitting, enabling us to achieve optimal model performance. The maximum number of epochs for training was set to 1000.

During the training phase, the model’s weights were updated using approximately 80% of the site data, and the remaining 20% were used to assess the performance of the model. The input sequence data for LSTM training were prepared by utilizing a sliding window approach. The dataset was composed of sequences with a fixed number of time steps, set at 23 steps. To ensure comparability across features, the dataset underwent normalization via the application of min–max scaling. The input sequences were then structured by concatenating historical data points and the features of the current time step, so that the LSTM model had access to comprehensive historical context. This approach ensured that each estimation was built upon prior context. A batch size of 16 was a rational trade-off to balance computational efficiency and training stability. This choice facilitated efficient parameter updates while maintaining training stability. Because the central objective of this study was to propose an innovative methodology for the estimation of solutes, the training procedures in our model were different for each site. Site 1 served predominantly as a foundation to establish the computational framework, whereas Site 2 allowed the purpose of assessing the efficacy and applicability of the introduced technique to be assessed. 

### 4.2. Input Selection

A total of 5016 distinct groups were meticulously created, encompassing a diverse range of input variables, ranging from 3 to 11, using measured and derived variables. The primary focus of this study was to maximize the likelihood while utilizing specific nuisance parameter sets (*α* and *β*) for different subsets of latent variables. As such, the penalty for the total number of parameters was not considered [41]; instead, emphasis was placed on enhancing the likelihood through the manipulation of latent variable subsets using ensemble averaging.

To ensure the reliability of our results, each group underwent a rigorous training and evaluation process. The neural network models were retrained and retested in 20 trials. This extensive exploration enabled a thorough examination of the impact of various input configurations on the performances of neural network models. The inclusion of multiple groups and repeated tests contributes to a comprehensive understanding of the intricate relationship between the input variables and the results obtained from the neural network models.

### 4.3. Model Evaluation

To evaluate the models, the mean absolute error (MAE), residual sum of squares (*RSS*), and coefficient of determination (*R*^2^) were used:

(6) $$MAE=\frac{\sum_{i=1}^{n}\left|A_{i}^{*}-A_{i}\right|}{n},$$

(7) $$RSS=\sum_{i=1}^{n}{\left(A_{i}^{*}-A_{i}\right)}^{2},$$

(8) $$R^{2}=1-\frac{RSS}{TSS},$$

where TSS is the total sum of squares. Additionally, the root mean square error (*RMSE*) was calculated from the *RSS* for later use.

(9) $$RMSE=\sqrt{\frac{RSS}{n}},$$

### 4.4. Use of Ensemble Averaging

In most cases, it is usual to select the best empirical model However, we propose the use of ensemble averaging as an alternative method to further enhance the precision of the prediction method. Ensemble operation, a widely applied technique to improve prediction accuracy, combines the output of multiple neural models [42]. Conventionally, ensembles encompass models constructed with varying neuron or layer configurations. However, our approach diverges by maintaining uniformity in the model architecture while embracing diverse input combinations across individual committees. In this study, we focus specifically on weighted averaging.

Weighted averaging assigns weights to each model based on performance instead of using uniform weights. Such an approach allows for a more flexible combination of model outputs, giving more weight to models demonstrating superior predictive capabilities. The predictions of each model are multiplied by their respective weights and then averaged to obtain the final prediction. For weight selection, we used the method proposed by A. Survyla et al. [33]:

(10) $$w_{i}=\frac{\sum_{j=1}^{n}{RMSE}_{j}-{RMSE}_{i}}{\sum_{j=1}^{n}{RMSE}_{j}\cdot (n-1)},$$

where the number of ensemble members is *n*.

## 5. Experimental Investigation

After training and validation using distinct groups from Site 1, the resulting models exhibited a range of mean absolute errors (MAE) of 0.2567 to 1.8496 g/L. The MAE values were obtained by averaging the results from 20 repeated tests, ensuring reliable assessments of the model performance. For the subsequent research, input groups with an MAE greater than 0.5 g/L were excluded. As a result, approximately 25% of the input groups remained for further analysis. The remaining groups were subjected to additional filtering; a group with a higher evaluation value was dropped if it had a subset identical to a group with a lower value. This stringent filtering process resulted in only 28 groups remaining, approximately 0.5% of the original input.

**Figure 2:**
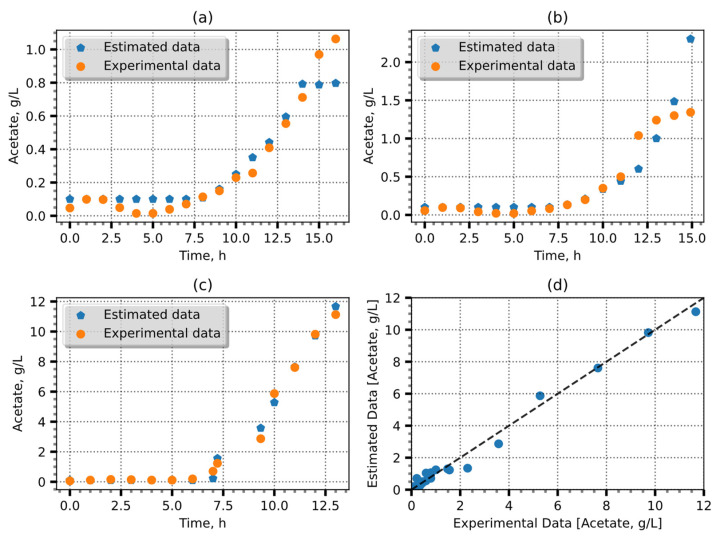
Acetate estimation using one RNN. (**a**–**c**) Three validation experiments and (**d**) comparison of estimates with observations, in which the black dashed line is the identity line.

The retained groups were then utilized to retrain the models, and the best models after 20 iterations were retained. The resulting models exhibited MAE values ranging from 0.1367 to 0.3375 g/L. The most accurate model predictions which had a coefficient of determination of −0.98 and an MAE of = 0.1367 or an error of 1.36% are shown in Figure 2.

**Figure 3:**
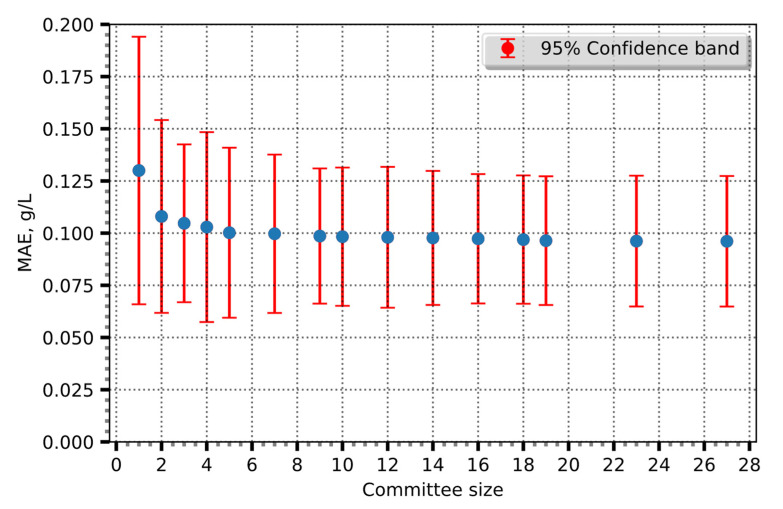
The correlation between MAE and committee size, with a 95% confidence band.

The disparities observed between the estimated and experimental data evident in the concluding segments of Figure 2a,b can be attributed to a discernible shift in the CPR measurements. In the scenario depicted in Figure 2a, a precipitous decline in the CPR value is evident, whereas, in Figure 2b, a corresponding abrupt escalation is observed. It is likely that these abrupt variations in the CPR are attributable to factors such as the accumulation of carbon dioxide (CO_2_) or other metabolic activities. Subsequently, an algorithm was employed to identify the optimal committee. Through weighted ensemble averaging between predictions, 14 committees with varying numbers of members were generated. The use of weighted ensemble averaging to predict acetate concentrations improved the precision of the approach, leveraging the collective knowledge. Furthermore, an analysis of the MAE dependency on the number of committee members revealed that increasing the number of the committee members improved the predictions. This indirect effect can be seen in Figure 3.

The graph demonstrates that the initial inclusion of ensemble members leads to substantial improvements in prediction accuracy. However, as more members are added, the gains in system precision become progressively smaller. Such a phenomenon suggests that the collective knowledge and diversity of the committee members reach a point of diminishing returns, after which further additions do not yield significant improvements in the prediction performance.

### 5.1. Information Gain Maximization

We used Shannon entropy, a measure of uncertainty or information content, to determine the optimal size of the committee [43]. The Shannon entropy equation is defined as follows:

(11) $$H\left(X\right)=-\sum_{x\in X}p\left(x\right){log}_{2}p(x),$$

where entropy is *H*, the input parameter is set as *X*, and the probability *p*(*x*) of an input parameter are present in the ensemble. We derived the probabilities of each input parameter by counting the occurrences of each input parameter within the committee and dividing the count by the total number of members. We also applied the local interpretable model-agnostic explanation (LIME)—method to calculate the probabilities for the second instance of entropy [44]. The LIME technique was adapted to provide explanations for each individual data point within our dataset, utilizing the models selected for the ensemble. Because the LSTM method was used, these features encompassed multiple instances within the input variables. For probability computation, we collected the first 20 features from each data point and then evaluated the occurrences of specific input variables. The resulting probabilities derived from Shannon entropy provided insights into the importance of each input parameter and its contribution to the overall model explanation.

**Figure 4:**
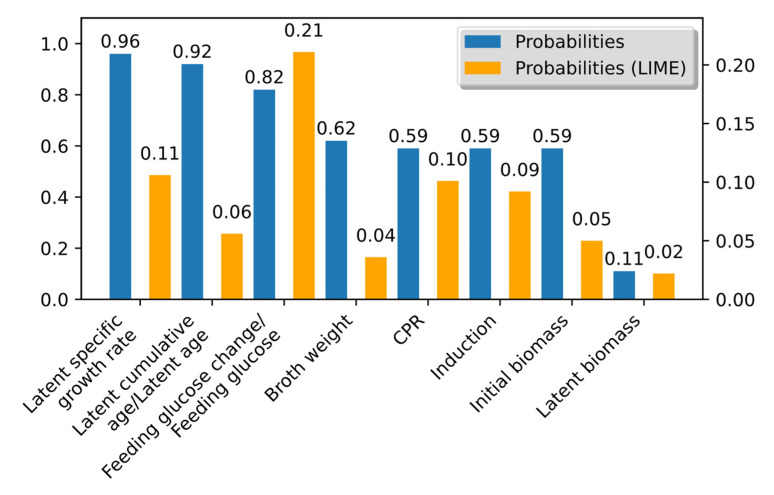
Appearance probability of model input in the ensemble.

As depicted in Figure 4, the latent variables, such as age, specific growth rate, or cumulative age, which exhibit higher probabilities indicate that these mathematical models offer a more comprehensive understanding of the entire process. The latent variables carry greater weight in explaining the dynamics and mechanisms of the modeled system, making Shannon entropy a powerful tool for assessing their significance and contribution to the overall model.

By applying Shannon entropy and evaluating its relationship with the committee size, meaningful committee members are identified, striking a balance between information capture and computational efficiency.

**Figure 5:**
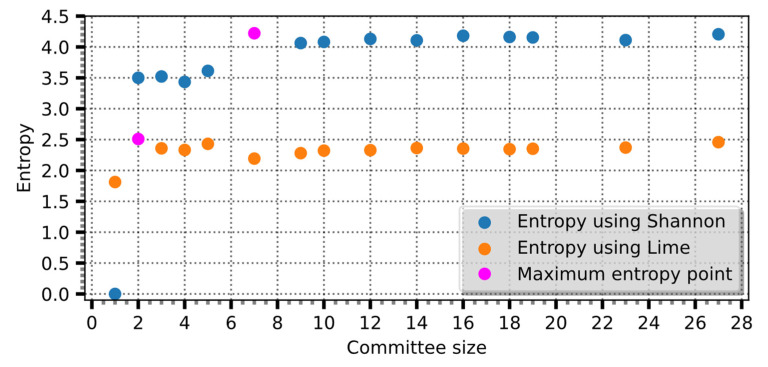
Entropy over the committee size, the orange color indicating the maximum entropy.

**Figure 6:**
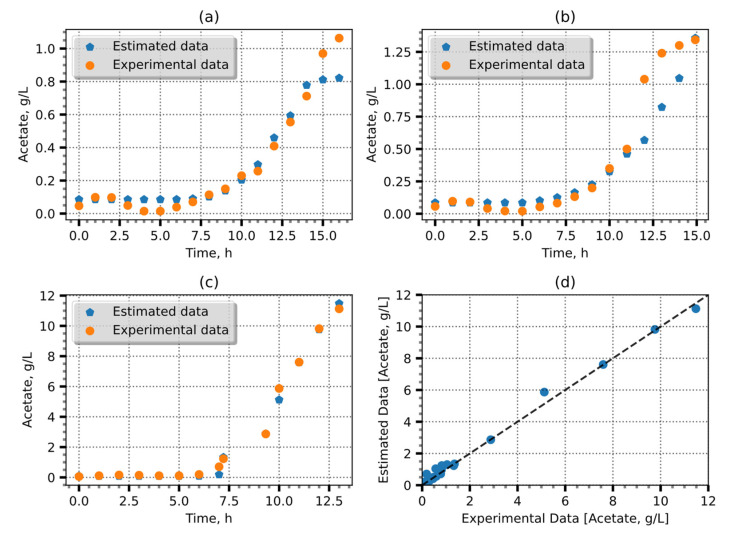
Acetate estimation using a committee size of seven. (**a**–**c**) Measurements ad estimates from three validation experiments over time; and chart (**d**) is the identity plot.

Our analysis, as shown in Figure 5, demonstrates that including more than seven members does not yield substantial additional information. On the other hand, use of the probabilities calculated by LIME suggests that a committee size of three yields the greatest information gain. Using ensemble size with maximum entropy improved the prediction by 27% and *R*^2^ - 0.99. The MAE of the improved model reached 0.098, and the overall error was 1% (Figure 6).

### 5.2. Hybrid Model Structure Evaluation

We conducted GSK data investigations to assess the performance of the proposed model structure. For data comparison, we employed the normalized mean absolute error (*nMAE*) as a quantitative metric [45], which offered a reliable measure of the precision of the model when applied to the data provided by GSK. The *nMAE* formula is defined as:

(12) $$nMAE=\frac{MAE}{A_{max}^{*}-A_{min}^{*}},$$

where the denominator values ($A_{max}^{*}$ and $A_{min}^{*}$) correspond to the minimum and maximum of the dataset. The *nMAE* provides a fair and consistent comparison across different parameters and sites.

**Figure 7:**
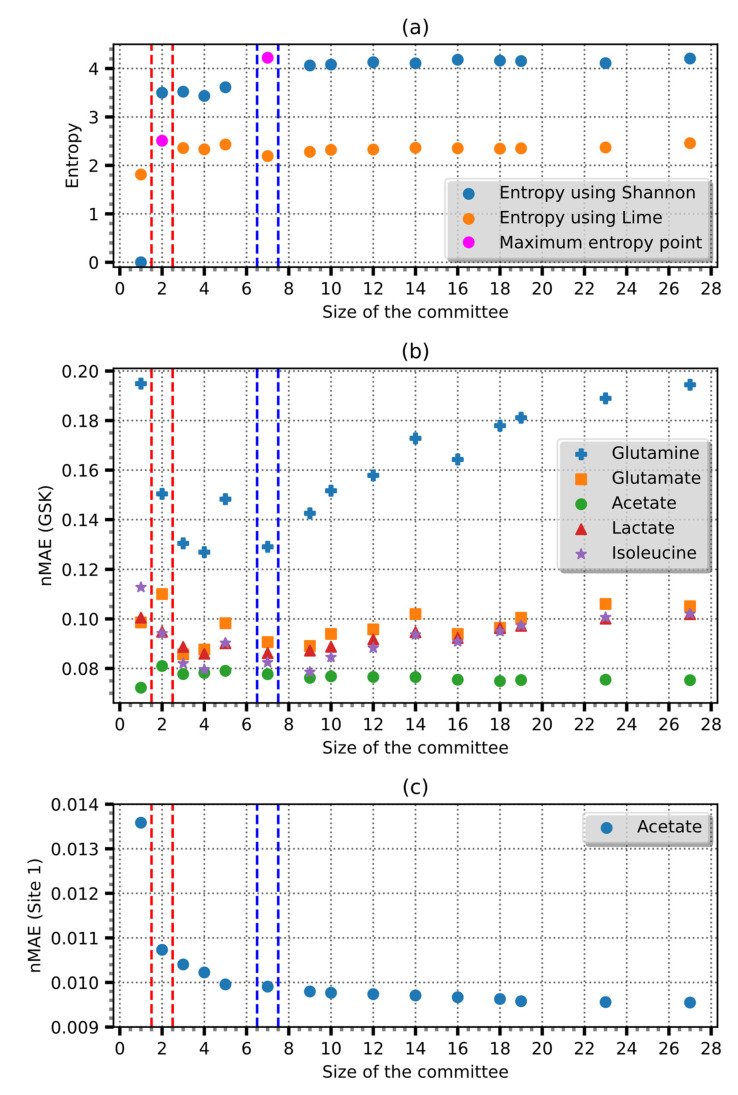
Comparison of the soluble compound estimation using GSK data. (**a**) The dependency of entropy on the committee size, whereas (**b**,**c**) the relationship between the normalized estimation (*nMAE*) and committee size. The dotted purple lines indicate the committee with the highest entropy using probabilities calculated from the input appearance in the committee ensemble, and the red dotted line shows the entropy calculated using the LIME method.

**Figure 8:**
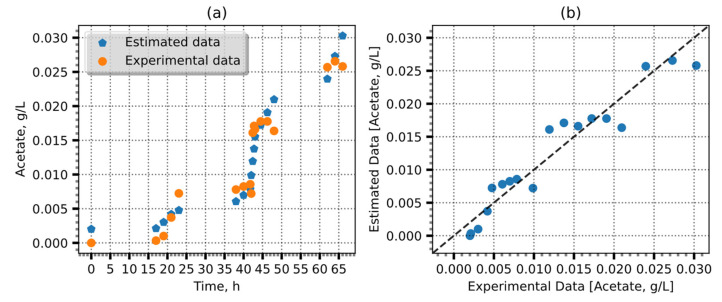
Acetate estimation using GSK data. (**a**) Estimates and observations over time. (**b**) Identity plot. The dotted line (**b**) shows perfect predictions and the blue dots—our model predictions.

Figure 7 demonstrates the relationship between the *nMAE* and the committee size used in the averaging of the ensemble, together with the errors in the estimates of the concentration of lactates, glutamate, glutamine, and isoleucine. The *nMAE* values generally decreased until they reached the maximum entropy; after which, they exhibited minimal variation or a slight increase. These findings validate the use of maximum entropy as a reliable criterion to determine the optimal number of committee members. Conversely, the application of maximum entropy derived using LIME revealed a distinct behavior, plateauing after the most significant error reduction and subsequently failing to manifest any further decline in error magnitude. Using data from GSK, the model achieved a *R*^2^ of 0.91 and MAE of 0.002 g/L or an error of 8% (Figure 8).

In addition, the inclusion of other metabolites helped to evaluate the performance of the model across multiple variables, providing valuable insights into the precision and versatility of the model.

**Figure 9:**
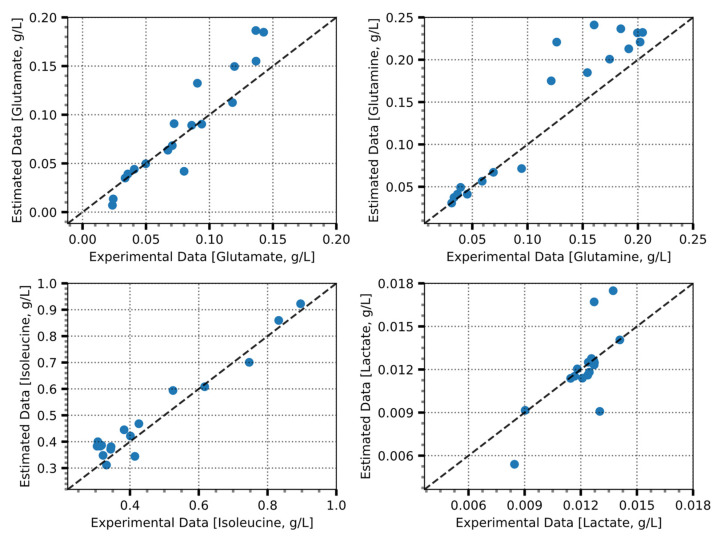
Additional plots with identity lines for glutamate, glutamine, isoleucine, and lactate concentrations.

The estimation results obtained using the hybrid model structure demonstrate its ability to estimate soluble compounds other than acetate. Although the errors were slightly more significant compared to the data from the first site (Table 4), this can be attributed to the limited dataset available from Site 2. Furthermore, accurate measurements of the small compound concentrations at site 2 were challenging because of the sensitivity limitations of the measurement techniques. However, the hybrid model exhibits promising potential to accurately estimate soluble compound concentrations in bioprocesses of *E. coli*. Additional plots with identity lines for other soluble compounds are shown in Figure 9.

## 6. Results and Discussion

In this study, we proposed a hybrid model structure and conducted an experimental investigation by selecting 5016 distinct input and latent variable combinations from Site 1. The initial models yielded MAE values ranging from 0.2567 to 1.8496 g/L. To refine the selection, input groups with an MAE greater than 0.5 g/L were excluded, reducing the set to 25% of its original size. Further rigorous filtering resulted in the retention of just 0.5% of the original groups, ultimately leading to a final set of 28 groups for further analysis. The MAE values of the final set of groups ranged from 0.1367 to 0.3375 g/L, with the best-performing model achieving an MAE of 0.1367 g/L, representing an error of 1.36%.

To improve the results, we used ensemble averaging. To optimize the ensemble size, we employed Shannon entropy method. Optimization revealed that an ensemble with seven members substantially enhanced the model precision (by 27%), leading to an MAE of 0.098 g/L, equivalent to a systematic error of 1%. Our devised methodology enhanced the flux method analysis by approximately 9% and in the extended Kalman filter by around 3% [6,21].

When evaluated using GSK data, our hybrid model displayed an acceptable level of precision, with an MAE of 0.002 g/L (8% error) for the estimation of acetate. Furthermore, the model exhibited promising capabilities for the estimation of other soluble compounds.

## 7. Conclusions

In conclusion, this research introduces a hybrid model structure for estimating concentrations of soluble compounds in *Escherichia coli* cultivation bioprocesses. The model effectively captures the dynamic processes inherent in such bioprocesses by leveraging off-gas data and essential physiological parameters through recurrent neural networks.

The incorporation of ensemble averaging, and information gain further enhances the precision of the estimation methods. In particular, the proposed hybrid model exhibits promising results, achieving estimations of the acetate levels with a low error rate of 1%. Extensive evaluations conducted using a dataset from two distinct sites highlighted the potential of the model for estimation of a diverse array of soluble compounds, with errors ranging from 8% to 13%.

The use of Shannon entropy identified that, the optimal committee size found for ensemble averaging was seven members, leading to a 27% improvement in model precision. The structure of the suggested model improved the results achieved in our previous work by approximately 36% [27]. The introduced approach provides a noninvasive and real-time method for monitoring bioprocesses involving *E. coli*.

## Figures and Tables

**Table 1:** Overview of soluble compounds estimation methods.

Model Type	Model Structure	Year	Soluble Compounds	Ref.
**Mechanistic** (model-driven)	Flux balance analysis	1994	Acetate	[6]
	Balance of biomass concentration, specific growth, and substrate uptake rate	1999		[7]
	Balances of biomass, glucose, lactose, acetate, and product concentrations			[8]
	Balance of liquid volume, biomass, glucose, acetate, and the dissolved oxygen concentrations	2001		[9]
	Balance equations	2010		[10]
	Kinetic model	2003		[11]
	Extended Calman Filter and Asymptotic Observers	2005	Lactate	[12]
	Pulse-based system	2018	Acetate	[13]
	Model parameters are changed depending on the event	2022	Ethanol	[14]
	Adaptive extended Luenberger observer	2015	Acetate	[15]
	Specific production rates and volume ratios	1999	Acetate, Lactate, Formate	[18]
	Indirect estimation using dissolved oxygen probe	2001	Acetate	[19]
	Data from high-performance liquid chromatography (HPLC)	2010		[20]
	EKF using information from biomass concentration, dissolved substrate, oxygen, and oxygen transfer rate	2012		[21]
	An asymptotic observer and an extended Kalman filter	2020	Ethanol	[22]
**Empirical** (data-driven)	RNN and LSTM	2021	Lactate	[16]
	Multilayer perceptron (MLP), Recurrent neural network (RNN), Long short-term memory (LSTM), Gated recurrent unit (GRU)	2023	Lactate	[17]
	Feed-forward and recurrent neural network	1992	Ethanol	[23]
	ANN and PLSR	2002	Gibberellic acid	[24]
	Radial basis neural network	2006	Ethanol	[25]
**Hybrid**	Mass Balances of O_2_ and CO_2_ and data reconciliation algorithm	2009	Ethanol, acetate, glycerol, ammonium	[27]
	Specific growth rate and age-related information with a decision tree model	2022	Acetate	[28]

**Table 2:** Experimental conditions at Site 1.

Condition	State	Condition	State
Bioreactor volume	7.0 L	Broth volume	3.7 L
Temperature	37.0 °C	pH	6.8
pO_2_	20.0%	Feeding start	at 5–7 h

**Table 3:** Experimental conditions at Site 2.

Condition	State	Condition	State
Bioreactor volume	20.0 L	Broth volume	9.0 L
Temperature	28.0 °C, 37.0 °C	pH	7.0
Airflow	20.0 NL/min	Feeding start	at 21 h

**Table 4:** Summary of the estimation errors for the additional soluble compounds.

Additional Soluble Compound	MAE, g/L	Error, %	*R* ^2^
Glutamate	0.016	9	0.82
Glutamine	0.027	13	0.79
Isoleucine	0.05	8	0.9
Lactate	0.001	9	0.52

## Data Availability

Not applicable.
